# Complement component C3a plays a critical role in endothelial activation and leukocyte recruitment into the brain

**DOI:** 10.1186/s12974-016-0485-y

**Published:** 2016-01-28

**Authors:** Fengjiao Wu, Qiang Zou, Xiaodan Ding, Dongyan Shi, Xingxing Zhu, Weiguo Hu, Lixin Liu, Hong Zhou

**Affiliations:** Department of Immunology, Nanjing Medical University, 140 Hanzhong Road, Nanjing, JS 210029 China; Department of Immunology, Chengdu Medical College, Chengdu, 610083 Sichuan China; Shanghai Cancer Center and Institute of Biomedical Science, Shanghai Medical College, Shanghai Cancer Center, Fudan University, Shanghai, 200032 China; Department of Pharmacology, College of Medicine, University of Saskatchewan, Saskatoon, Saskatchewan, S7N 5E5 Canada

**Keywords:** Complement, CNS inflammation, Intravital microscopy, Adhesion molecule, Leukocyte recruitment

## Abstract

**Background:**

The complement system is becoming increasingly recognized as a key participant in many neurodegenerative diseases of the brain. Complement-deficient animals exhibit reduced neuroinflammation.

**Methods:**

In the present study, we administered intracerebroventricularly lipopolysaccharide (LPS) to mimic local infection of the brain and investigated the role of key complement component C3 in brain vasculature endothelial activation and leukocyte recruitment. The degree of neutrophil infiltration was determined by esterase staining. Leukocyte-endothelial interactions were measured using intravital microscopy. Cerebral endothelial activation was evaluated using real-time PCR and Western blotting.

**Results:**

Neutrophil infiltration into the brain cortex and hippocampus was significantly reduced in C3^−/−^ mice and C3aR^−/−^ mice but not in C6^−/−^ mice. We detected markedly attenuated leukocyte-endothelial interactions in the brain microvasculature of C3^−/−^ mice. Accordingly, in response to LPS administration, the brain microvasculature in these mice had decreased expression of P-selectin, E-selectin, intercellular cell adhesion molecule 1 (ICAM-1), and vascular cell adhesion molecule 1 (VCAM-1). Depletion of C3 from the circulation also caused reduction in VCAM-1 and E-selectin expression and leukocyte recruitment, suggesting that C3 in the circulation contributed to brain endothelial activation. Furthermore, C3^−/−^ mice exhibited decreased leukocyte recruitment into the brain upon tumor necrosis factor-α (TNF-α) stimulation. C3a activated the phosphorylation of p38 mitogen-activated protein kinase (MAPK) and nuclear factor-κB (NF-κB) and induced the upregulation of VCAM-1 and ICAM-1 expression in murine primary cerebral endothelial cells in vitro.

**Conclusions:**

Our study provides the first evidence that C3a plays a critical role in cerebral endothelial activation and leukocyte recruitment during inflammation in the brain.

**Electronic supplementary material:**

The online version of this article (doi:10.1186/s12974-016-0485-y) contains supplementary material, which is available to authorized users.

## Background

Although the brain is considered to be an immune-privileged organ [1, 2], devastating effects can occur in the brain during local inflammation, particularly in response to cerebral bacterial infection. The recruitment of immune cells from the circulation is critical for many life-threatening central nervous system (CNS) inflammatory diseases, such as bacterial meningitis [3], multiple sclerosis [4], and stroke [5]. Neutrophils mobilized from the circulation in response to signals from the CXC family of chemokines, such as keratinocyte-derived chemokine (KC, CXCL1), and macrophage inflammatory protein-2 (MIP-2, CXCL2), are considered to be the first line of defense against bacteria [6–9]. Once activated through cytokines, such as tumor necrosis factor-α (TNF-α) and interferon-γ (IFN-γ), endothelial cells upregulate adhesion molecule expression, which enhances their interactions with the leukocytes and increases their transmigration into the brain [10, 11]. Neutrophils have been identified as the key players in CNS inflammation, and strategies that block neutrophil recruitment have been demonstrated as beneficial for the treatment of many types of CNS inflammation [12–14]. In a previous study, we showed that microglia play a dominant role in the innate immune response to infectious agents in the CNS [15]. Additionally, it has been widely accepted that inflammatory cytokines, such as TNF-α and chemokines from glial cells, activate the endothelium, thereby increasing adhesion molecule expression and leukocyte recruitment [16–18].

The complement system consists of approximately 40 soluble and membrane-bound proteins that play a central role in host defense against pathogens and inflammation initiation [19, 20]. Although the liver is the primary source of complement production, increasing evidence from recent studies has shown that many types of resident cells in the CNS produce complement components [21]. The expression of complement receptors, such as the C3a and C5a receptors on glial cells and neurons in the CNS, has been reported [22, 23]. Further studies in animal models have provided evidence for the involvement of the complement system in modulating CNS inflammation [24, 25]. In particular, systemic complement depletion reduces perihematomal brain edema and TNF-α production following experimental intracerebral hemorrhage [26]. Moreover, C3^−/−^ mice exhibited less brain edema and less microglial activation and neutrophil infiltration around the clot after intracerebral hemorrhage [27]. In a mouse model of meningitis induced by *Streptococcus pneumonia* infection, C1q and C3 deficiency led to reduced cerebrospinal fluid (CSF) leukocyte counts in comparison with infected wild-type (WT) mice [28]. Additionally, mice lacking C3 and its receptors developed increased burdens of West Nile virus in the CNS [29]. C5a receptor-deficient mice with pneumococcal meningitis also showed lower CSF leukocyte counts and alleviated brain damage compared with WT mice. Moreover, treatment with C5-specific monoclonal antibodies prevented animal death in WT mice with pneumococcal meningitis [30]. These studies suggest that the complement system plays a significant role in the CNS inflammatory response. However, the mechanisms underlying the contribution of complement components to immune cell recruitment into the brain parenchyma through the blood-brain barrier (BBB) remain unclear.

In this study, we systemically examined the detailed mechanisms underlying the contribution of complement components to leukocyte recruitment in response to cerebral lipopolysaccharide (LPS) administration. We first detected significantly reduced infiltrating neutrophils in the brain of C3-deficient (C3^−/−^) and C3a receptor-deficient (C3aR^−/−^) but not C6-deficient (C6^−/−^) mice. C3 deficiency also significantly reduced endothelial activation and leukocyte-endothelial interactions in brain postcapillary venules. Permeability changes in the BBB were comparable between WT and C3^−/−^ mice. Adhesion molecules, such as E-selectin and vascular cell adhesion molecule 1 (VCAM-1), showed reduced expression levels in the brains of C3^−/−^ mice in response to LPS administration, suggesting that C3 contributes to the activation of brain endothelium. Furthermore, TNF-α was not able to induce leukocyte recruitment in C3^−/−^ mice. C3a directly stimulated cerebral endothelial activation in vitro. Taken together, these results demonstrate that C3a plays a critical role in endothelial activation and subsequent leukocyte recruitment in the brain in response to intracerebroventricular LPS administration.

## Methods

### Animals

Adult male wild-type (WT) C57BL/6J and BALB/c mice 7 to 8 weeks old weighing 20 to 25 g were obtained from the Model Animal Research Center, Nanjing University. C3-deficient (C3^−/−^) mice (C57BL/6J mice background) and C3a receptor-deficient (C3aR^−/−^) mice (BALB/c mice background) were purchased from the Jackson Laboratory (Bar Harbor, ME, USA). The C5aR^−/−^ mice (C57BL/6J background) were obtained from Dr. Hu Weiguo (Shanghai Cancer Center and Institute of Biomedical Science, Shanghai Medical College, Fudan University, China). The C6-deficient (C6^−/−^) mice (C57BL/6J mice background) were a gift from Dr. Tod Merkel (Center for Biologics Evaluation and Research, Food and Drug Administration, USA). The animals were housed under a 12-h light/dark cycle under specific pathogen-free conditions with free access to food and water. All animal protocols were reviewed and approved by the Institutional Animal Care and Use Committee of Nanjing Medical University. All animal experiments were carried out in a blinded and randomized fashion.

### Intracerebroventricular LPS injection

The mice were anesthetized using an intraperitoneal (i.p.) injection of 200 mg/kg of ketamine and 10 mg/kg of xylazine. Subsequently, the mice were placed onto a rodent stereotaxic frame (David Kopf Instruments, Tujunga, CA, USA). The scalp was shaved, and a burr hole was drilled 1 mm caudal to the bregma and 2.0 mm lateral to the midline [15, 31]. A total of 2 μL of 2 μg of LPS (*Escherichia coli* serotype 0111:B4 strain; InvivoGen) or 0.2 μg of TNF-α (R&D systems, Minneapolis, MN, USA) was administered by intracerebroventricular (i.c.v.) injection using a 10-μl Hamilton microsyringe over a 3-min period. Control animals received an isovolumetric i.c.v. injection of saline. Body temperature was monitored using a rectal probe, and the mice were maintained under deep anesthesia at 36 ± 1 °C using a thermostatic heating system (Harvard Apparatus, MA, USA) throughout the experiment.

### Immunohistochemical procedure

Mice under deep anesthesia and received i.c.v. LPS or saline injection were perfused through the heart with ice-cold 4 % formalin. The cerebral tissues were then removed and fixed in 4 % formalin for 48 h. Thick coronal sections were obtained at −1.0 to −3.0 mm from the bregma. Formalin-fixed tissues were embedded in paraffin and subsequently sectioned at a 4-μm thickness using a cryostat. Infiltrating neutrophils were detected using a Naphthol AS-D Chloroacetate-Specific Esterase kit (Sigma-Aldrich, St. Louis, MO, USA).

### Intravital microscopy

The animals were anesthetized and monitored as described previously [15]. A craniotomy was performed using a high-speed drill in the right parietal bone. Stripping the dura from the site exposed the brain pial vessels. Subsequently, the animals were intravenously (i.v.) administered rhodamine 6G (Sigma-Aldrich) (0.5 mg/kg, body weight) to label leukocytes. Leukocyte-endothelial interactions were recorded using an sCMOS camera (ORCA-Flash 4.0, HAMAMATSU) mounted onto a Nikon FN1 microscope. Three different postcapillary venules with diameters between 30 and 70 μm were chosen for observation. All experiments were recorded for subsequent playback analysis. Rolling leukocytes were defined as cells moving at a velocity less than that of erythrocytes. Cells remaining stationary for at least 30 s were considered adherent.

### Blood-brain barrier permeability determination

The levels of albumin, a plasma protein that is normally excluded from the brain by the intact BBB, were used as an indicator for its integrity. The mice were anesthetized and perfused with 20 ml of ice-cold phosphate-buffered saline (PBS) to remove contaminated albumin from the circulation. Then, the concentration of albumin in brain homogenates was measured by Western blotting as previously described [32].

### Complement depletion

Four hours prior to i.c.v. LPS injection, hypocomplementemia was induced through the i.p. injection of cobra venom factor (CVF) (Biogen Sci & Tech Co., Kunming, China) in PBS into C57BL/6J mice at a dose of 10 μg/mouse (15 U/mouse). This treatment depletes complement within 4 h of the injection, reducing the levels of C3 in the circulation to less than 3 % of the normal range, which persists for at least 48 h post-injection [33, 34].

### RNA isolation and real-time quantitative PCR

Total RNA was extracted from brain tissue using Trizol reagent (Invitrogen, Carlsbad, CA, USA) and was then reversely transcribed using Superscript II (Invitrogen, Carlsbad, CA, USA). Real-time PCR was performed using a SYBR green PCR reagent according to the manufacturer’s instructions (Applied Biosystems, Yerevan, Armenia). The following DNA sequences were used for the primer pairs: P-selectin forward primer, 5'-TCCAGGAAGCTCTGACGTACTTG-3'; P-selectin reverse primer, 5'-GCAGCGTTAGTGAAGACTCCGTAT-3'; E-selectin forward primer, 5‘-TGAACTGAAGGGATCAAGAAGACT-3'; and E-selectin reverse primer, 5'-GCCGAGGGACATCATCACAT-3'; VCAM-1 forward primer, 5‘-TGACAAGTCCCCATCGTTGA-3'; and VCAM-1 reverse primer, 5'-ACCTCGCGACGGCATAATT-3'; intercellular cell adhesion molecule (ICAM-1) forward primer, 5‘-CCTGTTTCCTGCCTCTGAAG-3'; and ICAM-1 reverse primer, 5'-GTCTGCTGAGACCCCTCTTG-3'. Mouse β2-microglobulin (β_2_-MG) was used as an internal control with the following primer sequences: β_2_-MG forward primer, 5'-CCTGCAGAGTTAAGCATGACAGT-3'; and β_2_-MG reverse primer, 5'-TCATGATGCTTGATCACATGTCT-3'. Quantitative PCR was performed with an ABI Prism 7300 spectrofluorometric thermal cycler (Applied Biosystems) using SYBR Green I as a double-stranded DNA-binding dye. The amplification conditions consisted of 95 °C (2 min), followed by 32 cycles of 95 °C (20 s), 57.2 °C (30 s), and 72 °C (30 s). Quantitative PCR assays were conducted in triplicate and were then quantitated using the 2^−ΔΔCt^ method. The data are expressed as *n*-fold differences relative to the calibrator.

### Enzyme-linked immunosorbent assay

The mice were anesthetized after LPS injection and subsequently perfused through the heart with cold PBS to remove blood proteins from the circulation. The brains were rapidly removed and homogenized in 1 ml of sterile PBS, followed by centrifugation at 12,000 rpm for 5 min at 4 °C. The supernatants were assayed for determining TNF-α and IL-1β concentrations using commercial enzyme-linked immunosorbent assay (ELISA) kits (TNF-α, BD, San Diego, CA, USA; IL-1β, eBioscience, San Diego, CA, USA) according to the manufacturers’ instructions. To measure the levels of C3a in brain and plasma of mice, plates were coated with 100 μl of rat anti-mouse capture antibody specifically against C3a (BD Biosciences, San Jose, CA, USA) at 1:250 dilutions in PBS overnight at 4 °C. FUT-175 (BD Biosciences) which was dissolved with 1 ml ddH_2_O, a synthetic inhibitor for the classical and alternate pathways of complement activation, was added into the EDTA blood and brain samples according to the manufacturer’s instructions. In details, 10 μl of FUT-175 was added into 1 ml of freshly drawn EDTA blood or brain supernatant on ice. After centrifugation, the plasma and brain proteins in these samples were collected for the ELISA assay. Brain, plasma samples, or C3a standard solution were diluted and added to the plates coated with capture antibody and incubated at room temperature for 2 h, and purified mouse C3a protein (BD Bioscience) was set as standard. Brain and plasma samples from C3^−/−^ mice were set as negative control. After washing four times, 100 μl of biotin-conjugated rat anti-mouse C3a (BD Biosciences) at 1:500 dilution in PBS with 10 % FBS was added to each well and incubated at room temperature for 1 h. The wells were washed again and then incubated with a 1:250 dilution of streptavidin/horseradish peroxidase (HRP) (BD Biosciences) for 1 h at room temperature. The wells were washed, and 100 μl of substrate solution (BD Biosciences) was added to each well. The color was developed for 10–20 min with the reaction stopped by the addition of 2N H_2_SO_4_. Absorbance was read at 450 nm with correction for absorbance at 550 nm.

### Western blotting

The complement factor C3 and albumin were analyzed in the brain homogenates and plasma samples obtained from experimental mice. To obtain the brain homogenates, the mice were anesthetized at 4, 12, and 24 h after i.c.v. LPS injection and subsequently perfused with ice-cold PBS to clear blood-borne proteins. Each brain was removed and homogenized in PBS on ice, followed by centrifugation at 12,000 rpm for 5 min. For the analysis of plasma complement content, blood (100–400 μl) was obtained by intracardiac puncture of anesthetized animals immediately prior to the perfusion. 10 μl of FUT-175 (Futhan) was immediately added into each milliliter of the freshly drawn EDTA blood on ice. After centrifugation at 2500 rpm for 10 min, these plasma samples were boiled in loading buffer for another 10 min. Subsequently, the samples were diluted and separated on a 10 % acrylamide-sodium dodecyl sulfate (SDS) gel, followed by transferring onto membranes and blotting overnight at 4 °C with the following antibodies: anti-C3 (Abcam, Cambridge, USA), anti-albumin (Abcam), anti-E-selectin (Abcam), anti-VCAM-1 (Abcam), anti-ICAM-1 (Abcam), anti-GAPDH (Abcam), anti-NF-kB p65 (Cell Signaling Technology, Beverly, CA, USA), anti-phospho-NF-κB p65 antibody (Cell Signaling Technology), and anti-β-actin (Cell Signaling Technology). The membrane was washed (0.05 % Tween-20 in PBS), incubated with peroxidase-labeled goat anti-rabbit IgG or anti-goat IgG, and washed again. Antibody binding was visualized using enhanced chemiluminescence reagents (PerkinElmer, Waltham, MA, USA). Densitometric images were quantified using ImageJ software (National Institutes of Health, Bethesda, MD, USA). Relative expression levels of proteins were normalized to β-actin.

### Isolation and culture of murine cerebral endothelial cells

The mice were sacrificed, and their brains were collected. The cerebral cortices, devoid of cerebella, white matter, and leptomeninges, were minced into small pieces. The pellet was digested in 15 ml of 0.1 % collagenase B (Roche, Indianapolis, IN, USA) supplemented with 30 U/ml of DNase I (Sigma-Aldrich) for 1.5–2 h at 37 °C with occasional agitation. The microvessels were isolated through gradient centrifugation on 15 % dextran (Sigma-Aldrich) and subsequently digested in 0.1 % collagenase/dispase (Roche) supplemented with 20 U/ml of DNase I for 1.5–2 h at 37 °C with occasional agitation. The microvessel pellets were resuspended in medium supplemented with 3 ng/ml of bovine fibroblast growth factor (bFGF, Peprotech, Rocky Hill, NJ, USA), 30 % fetal bovine serum (FBS), 10 U/ml of heparin, 100 U/ml of penicillin, and 100 mg/ml of streptomycin. The microvessel suspension was plated onto 6-well plates coated with rat-tail collagen I (Sigma-Aldrich) and incubated at 37 °C with 5 % CO_2_. The medium was changed every 2 days. The endothelial cells began migrating from the vessels within 2–3 days and grew to confluence within 7–10 days.

### Statistical analysis

Statistical analysis was performed using SPSS software (17.0 for Windows, IBM Inc., Chicago, IL, USA). The values are expressed as the means ± standard errors of the mean (SEM). Differences between the two groups were analyzed using Student’s *t* test, and *P* < 0.05 was considered significant.

## Results

### C3 deficiency decreases LPS-induced neutrophil recruitment into the brain

**Fig. 1:**
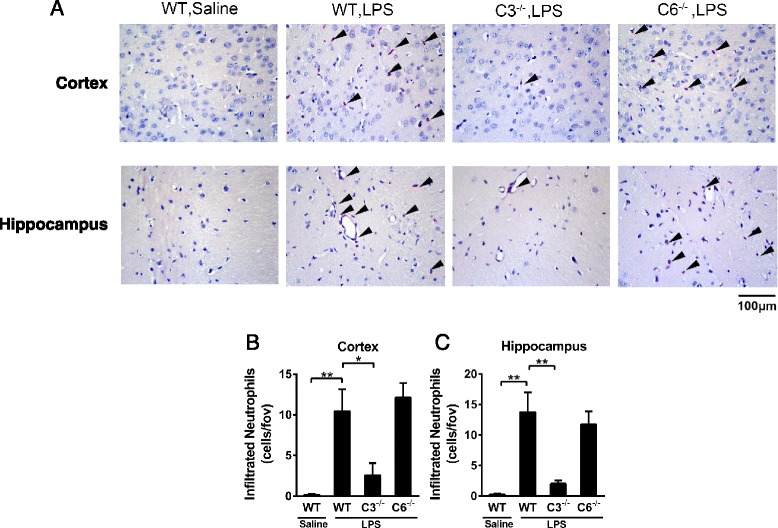
Effects of C3 and C6 deficiency on neutrophil recruitment into the brain parenchyma after i.c.v. LPS injection. **a** WT C57BL/6J mice (*n* = 8), C3^−/−^ mice (*n* = 8), and C6^−/−^ mice (*n* = 4) received i.c.v. injections of saline or LPS. After 24 h, esterase staining was performed to quantitate the neutrophils infiltrating into the cortex and hippocampus. LPS i.c.v. injection induced substantial infiltrations of neutrophils into the cortex and hippocampus of WT C57BL/6J mice. C3 deficiency significantly reduced neutrophil infiltration into the cortex and hippocampus. C6 deficiency did not reduce neutrophil recruitment. The quantitative data of infiltrated neutrophils in the cortex (**b**) and hippocampus (**c**) are shown as the means ± SEM. Magnification, ×400. *P < 0.05, **P < 0.01

To examine the roles of complement components in neutrophil recruitment into the CNS, WT C57BL/6J mice, C3^−/−^ mice, and C6^−/−^ mice were subjected to i.c.v. LPS injection. Brain sections from these mice were stained with esterase to evaluate neutrophil recruitment. In response to LPS, neutrophils initiated extravasation into the brain at 12 h post-injection, and infiltration peaked at 24 h in the cortex and hippocampus and subsequently decreased. Compared with WT mice, the number of neutrophils infiltrated into the brain cortex and hippocampus was significantly reduced in C3^−/−^ but not C6^−/−^ mice (Fig. 1a–c).

**Fig. 2:**
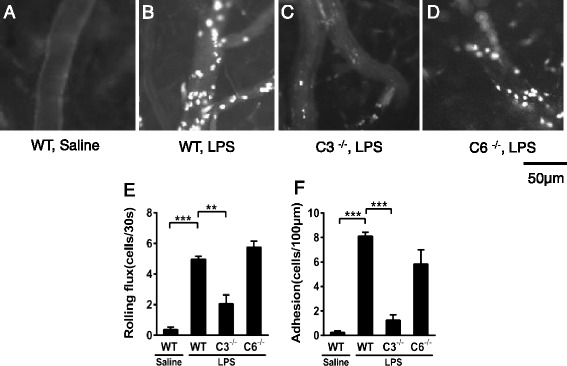
Leukocyte-endothelial interactions in WT and C3^−/−^ and C6^−/−^ mice after i.c.v. LPS injection. WT C57BL/6J mice and C3^−/−^ and C6^−/−^ mice received i.c.v. injections of LPS or saline, and leukocyte-endothelial interactions were examined through intravital microscopy. **a** WT C57BL/6J mice with saline injection showed no evident recruitment into CNS vessels. After i.c.v. injection of LPS, WT C57BL/6J mice with LPS injection showed significant recruitment into CNS vessels (**b**), and C3^−/−^ mice exhibited reduced recruitment into the CNS (**c**). **d** C6^−/−^ mice exhibited leukocyte-endothelial interactions comparable with those observed in WT mice. The numbers of rolling (**e**) and adhesive cells (**f**) are shown as the means ± SEM; *n* = 6 for all groups, **P < 0.01, ***P < 0.001

Using intravital microscopy, we further examined the roles of complement components in leukocyte-endothelial interactions in postcapillary venules in the CNS. The i.c.v. administration of saline did not induce leukocyte rolling and adhesion in brain postcapillary venules of WT mice (Fig. 2a). The i.c.v. administration of LPS induced significant leukocyte rolling and adhesion in brain postcapillary venules of WT mice (Fig. 2b, Additional file 1). Consistent with the immunohistochemical results, leukocyte recruitment in brain vessels was significantly reduced in the brain venules of C3^−/−^ (Fig. 2c, Additional file 2) but not C6^−/−^ mice (Fig. 2d, Additional file 3) at 4 h post-injection. Rolling (Fig. 2e) and adherent leukocytes (Fig. 2f) were quantified in WT, C3^−/−^, and C6^−/−^ mice. Taken together, these results suggest that neutrophil recruitment induced through i.c.v. LPS administration requires C3 but not C6.

### C3 deficiency decreases LPS-induced brain endothelial adhesion molecule expression but not permeability changes

**Fig. 3:**
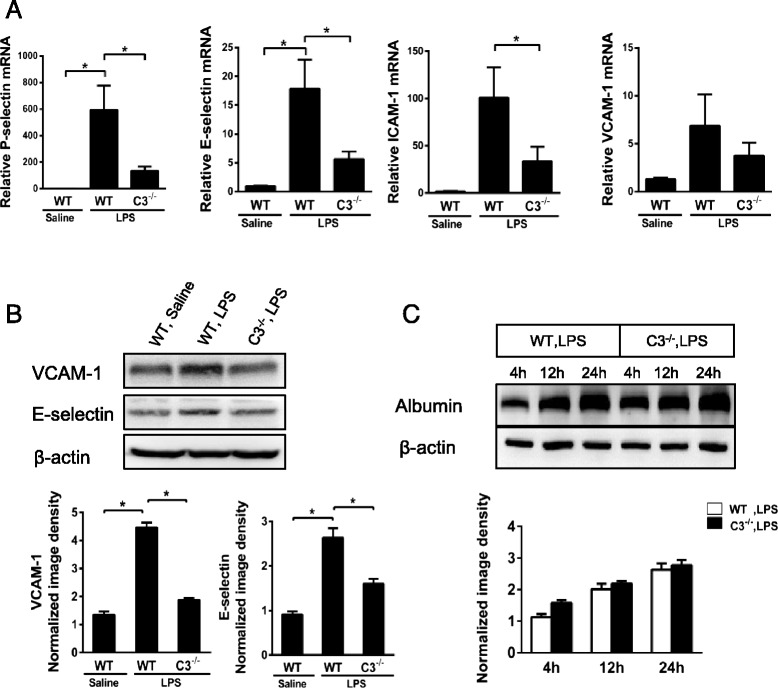
Effect of C3 deficiency on the expression of endothelial adhesion molecules and the BBB permeability changes in the brain. WT C57BL/6J mice and C3^−/−^ mice received i.c.v. injections of LPS; 4 h later, mRNA expression in brain tissue was quantified using real-time PCR. **a** C3 deficiency resulted in the downregulation of brain P-selectin, E-selectin, ICAM-1, and VCAM-1 mRNA expression (*n* ≥ 8). **b** VCAM-1 and E-selectin protein levels were measured through Western blotting analysis; the ratio of VCAM-1 and E-selectin to β-actin was determined through densitometry (*n* = 5). **c** To assess BBB permeability, mouse brain homogenates were examined for albumin infiltration using Western blotting analysis (*n* = 6). Albumin levels in the brain proteins were measured at 4, 12, and 24 h after LPS i.c.v. The results are shown as the means ± SEM; *P < 0.05

Adhesion molecules primarily mediate leukocyte-endothelial interactions and are markers of endothelial activation in acute inflammation [35, 36]. To determine whether C3 deficiency influenced endothelial activation, we examined the mRNA expression levels of different adhesion molecules upon LPS injection in C3^−/−^ mice. C3 deficiency resulted in significant reduced P-selectin, E-selectin, ICAM-1, and VCAM-1 transcription in the brain at 4 h after LPS injection (Fig. 3a). Moreover, Western blotting analysis also revealed the reduced expression of VCAM-1 and E-selectin in the brain of C3^−/−^ mice (Fig. 3b).

BBB permeability profoundly affects leukocyte recruitment in the brain. To examine whether C3 deficiency led to altered BBB permeability, we measured the brain concentrations of albumin leaked from the circulation into the brain tissues in LPS-treated WT and C3^−/−^ mice. The i.c.v. injection of LPS induced time-dependent increases in BBB permeability. However, there was no significant difference in the permeability between WT and C3^−/−^ mice (Fig. 3c). Taken together, these data suggest that reduced endothelial adhesion molecule expression caused by lower grade of endothelial activation, but not the altered brain BBB permeability, led to the reduced neutrophil recruitment in C3^−/−^ mice. Clearly, C3 deficiency affected the activation of the brain endothelium upon LPS challenge.

### C3 depletion from circulation affected leukocyte recruitment in the brain

**Fig. 4:**
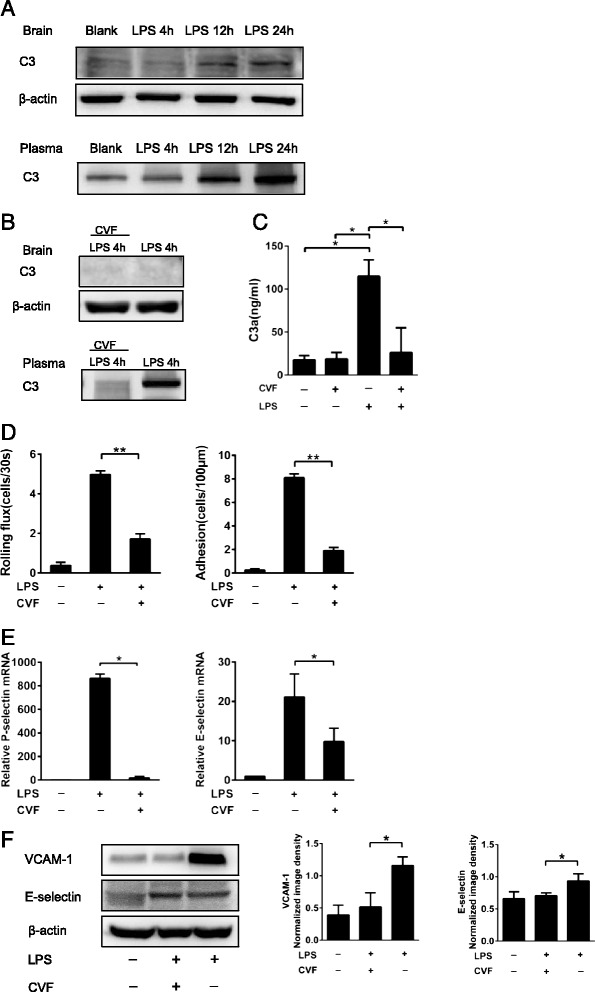
Effects of complement depletion on leukocyte recruitment into the brain parenchyma after i.c.v. LPS injection. **a** WT C57BL/6J mice were treated i.c.v. with LPS, and C3 levels in the brain (without dilution, cleared from blood proteins) and plasma (diluted in 1:100) were determined by Western blotting. **b** WT C57BL/6J mice received i.p. injections of CVF (10 μg/mouse) or PBS. After 4 h, WT C57BL/6J mice (*n* = 4) received i.c.v. LPS injection, and after 4 h, plasma and brain samples were then collected for C3 Western blotting analysis. At 4 h after CVF injection, WT C57BL/6J mice (*n* = 4) received i.c.v. LPS injection, and plasma samples were collected for C3a ELISA assay (**c**). At 4 h after i.p. injection of CVF to deplete complement from the blood circulation, mice received i.c.v. LPS injection. Then, after 4 h, rolling and adhesion leukocytes were quantified using intravital microscopy (**d**). **e** P-selectin and E-selectin mRNA expressions were measured using real-time PCR. **f** VCAM-1 and E-selectin protein levels in the brain were examined by Western blotting (*n* = 4). Images are representative of four experiments. The ratio of VCAM-1 and E-selectin to β-actin was determined through densitometry. The results are shown as the means ± SEM. *P < 0.05, **P < 0.01; *n* ≥ 4 for all groups

Virtually all complement components can be locally produced in the CNS in response to injury or inflammation [21]. To determine the source of C3, which plays a major role in endothelial activation, we assessed the C3 levels in the CNS and blood circulation. C3 was virtually undetectable in the brain parenchyma at 4 h post-i.c.v. injection of LPS. Thereafter, C3 levels in the brain parenchyma progressively increased from 4 to 24 h, peaking at 24 h after LPS injection (Fig. 4a). The plasma levels of C3 remained stable in the first 12 h and thereafter increased progressively from 12 to 24 h after LPS treatment (Fig. 4a). Notably, the C3 levels in the circulation were significantly higher than those in the CNS, even after a 100-fold dilution of the plasma sample (Fig. 4a).

We examined leukocyte recruitment in mice at 4 h after receiving i.c.v. injection of LPS when C3 levels were depleted from the circulation by using CVF. CVF treatment depleted 95 % of the C3 in circulation after 4 h (Fig. 4b). However, C3 levels in the brain at 4 h after the CVF treatment were very low and not affected by CVF (Fig. 4b). The plasma levels of C3a were increased significantly at 4 h after LPS treatment. CVF treatment depleted the majority of C3a in these LPS-treated animals (Fig. 4c). C3 depletion from the circulation significantly reduced rolling and adhesion (Fig. 4d) of leukocytes in brain vessels. Furthermore, CVF treatment significantly reduced the levels of LPS-stimulated P-selectin and E-selectin (Fig. 4e) mRNA expression in the brain. Additionally, CVF treatment reduced the protein levels of VCAM-1 significantly, and also reduced E-selectin slightly in LPS-treated mouse brains (Fig. 4f).

### C3a and C3aR participate in leukocyte recruitment into the cerebral microvessels

**Fig. 5:**
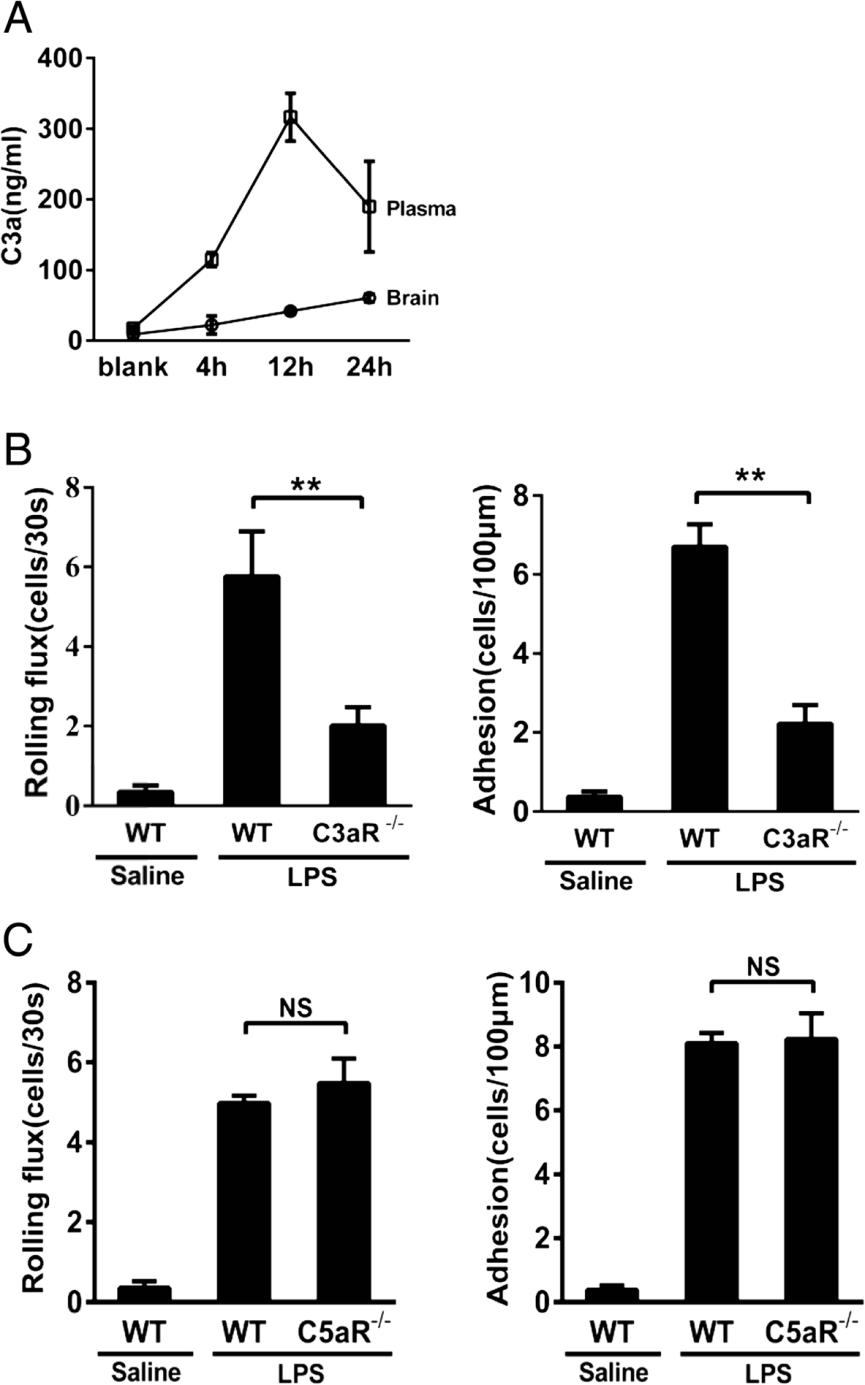
The C3a receptor is essential for LPS-induced leukocyte recruitment into the brain vasculature. **a** WT C57BL/6J mice were i.c.v.-treated with LPS, and C3a levels in brain and plasma were then measured at 0, 4, 12, and 24 h after LPS injection by ELISA. WT BALB/c mice, C3aR^−/−^ mice, WT C57BL/6J mice, and C5aR^−/−^ mice were i.c.v.-treated with LPS, and 4 h later, intravital microscopy was performed. Leukocyte rolling flux and adhesion (**b**–**c**) are shown as the means ± SEM. **P < 0.01; *n* ≥ 4 for all groups

C3a, the cleavage product of C3, is an anaphylatoxin that triggers an inflammatory response engaging many types of cells to release cytokines and other inflammatory mediators. Upon i.c.v. LPS administration, the levels of C3a in the plasma were gradually increased at 4 h and substantially further increased at 12 and 24 h (Fig. 5a). In contrast, the C3a level in the brain parenchyma remained only marginally increased during this period (Fig. 5a). C3a acts on the receptor C3aR to exert its biological functions [37, 38]. Using intravital microscopy, we further observed that LPS i.c.v. injection caused very limited rolling and adhesive (Fig. 5b) leukocytes in cerebral vasculature of C3aR^−/−^ mice compared to the WT BALB/c mice. Intravital microscopy revealed similar levels of leukocyte-endothelial interactions in LPS-treated C5aR^−/−^ mice, as compared to LPS-treated WT C57BL/6J mice (Fig. 5c). These results further confirmed that C3a and C3aR, but not C5a or C5aR, is essential for endothelial activation and subsequent leukocyte-endothelial interactions in the brain inflammation.

### C3 was essential for TNF-α-induced cerebral endothelial-leukocyte interactions in vivo

**Fig. 6:**
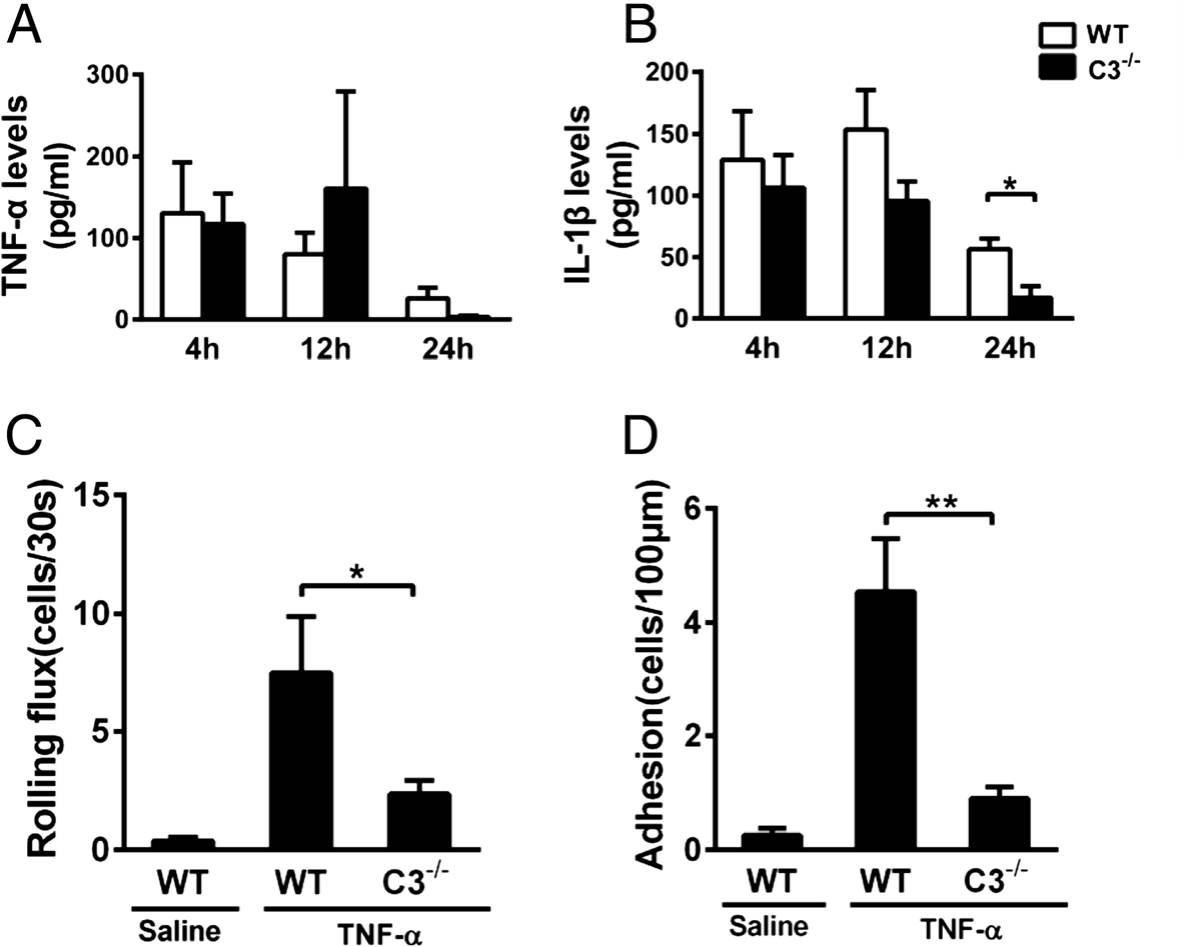
TNF-α has a synergistic effect with C3 on the induction of leukocyte recruitment in vivo. C3^−/−^ mice and WT C57BL/6J mice were treated i.c.v. with LPS, and TNF-α (**a**) and IL-1β (**b**) concentrations in the brain extracts were then measured using ELISA. WT C57BL/6J mice and C3^−/−^ mice were treated i.c.v. with TNF-α (0.2 μg), and intravital microscopy was performed to examine leukocyte recruitment in WT C57BL/6J mice and C3^−/−^ mice at 4 h after i.c.v. TNF-α injection. The data for leukocyte rolling flux (**c**) and adhesion (**d**) are shown as the means ± SEM. *P < 0.05, **P < 0.01; *n* ≥ 4 for all groups

To determine whether the reduced leukocyte recruitment observed in C3^−/−^ mice was indicative of defective inflammatory cytokine production, we measured the levels of the inflammatory cytokines TNF-α and IL-1β in the brain of the mice i.c.v.-treated with LPS. Interestingly, the TNF-α levels in the brain were comparable in WT and C3^−/−^ mice (Fig. 6a, *P* > 0.05), whereas the IL-1β levels were significantly reduced only after 24 h in C3^−/−^ mice (Fig. 6b).

High levels of inflammatory cytokines (Fig. 6a, b) and chemokines (data not shown) were detected in the brain, while cerebral endothelial cells were not fully activated in the absence of C3. Furthermore, we administered i.c.v. injections of TNF-α into C3^−/−^ mice and observed that leukocyte rolling and adhesion were significantly reduced compared with WT mice (Fig. 6c, d). This result suggests that there may be a synergistic effect of C3 with TNF-α on brain endothelial cell activation in vivo.

### C3a induces brain endothelial cell activation and adhesion molecule expression

**Fig. 7:**
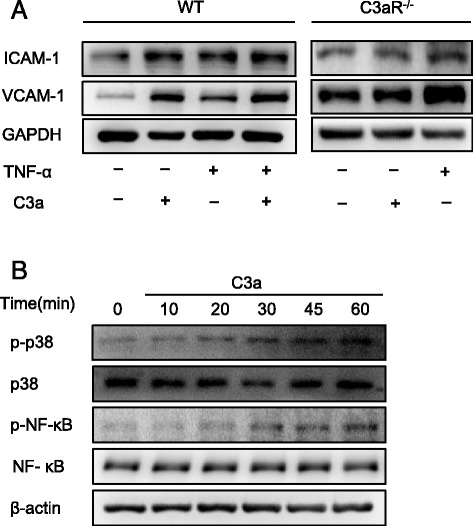
C3a stimulates primary brain endothelial activation and signaling pathways in vitro. Primary brain microvascular endothelial cells of WT C57BL/6J mice and C3aR^−/−^ mice (**a**) were stimulated with either vehicle, C3a (200 ng/ml), TNF-α (1 ng/ml), or C3a (200 ng/ml) plus TNF-α (1 ng/ml) for 4 h, and the cell lysates were then analyzed for the expression of adhesion molecules using Western blotting. **b** Primary brain endothelial cells from wild-type mice were incubated with either vehicle or C3a for the indicated time points, and phosphorylated forms of p65 NF-κB and the MAPK p38 in cell lysates were then detected using Western blotting. Images are representative of four experiments

To further confirm the role of C3a in cerebral endothelial activation, primary brain endothelial cells were isolated from WT C57/BL6J mice and treated with C3a (200 ng/ml) and TNF-α (1 ng/ml) for 4 h. As expected, TNF-α stimulated the increased ICAM-1 and VCAM-1 expression in brain endothelial cells from WT mice. C3a induced ICAM-1 and VCAM-1 expression in brain endothelial cells from WT mice but not C3aR^−/−^ mice (Fig. 7a). Further, C3a (200 ng/ml) and TNF-α (1 ng/ml) have a synergistic effect on inducing the expression of ICAM-1 and VCAM-1 in primary cerebral endothelial cells (Fig. 7a). In addition, C3a induced the phosphorylation of p38 mitogen-activated protein kinase (MAPK) and NF-κB in brain endothelial cells from the WT mice (Fig. 7b). Therefore, C3a stimulates the expression of adhesion molecules in primary cerebral endothelial cells in vitro.

## Discussion

The brain has long been considered to be an immune-privileged organ. However, it is well appreciated that the brain can respond to pathogens and danger signals; in particular, resident glial cells generate inflammatory cytokines and chemokines [39, 40], which in turn activate endothelial cells to recruit immune cells into the brain [15]. In CNS inflammation, elevated levels of C3 in CSF in humans and mice with bacterial meningitis have been previously reported [41, 42]. In addition, several studies have shown critical roles for complement components in brain inflammation and reduced leukocyte recruitment in complement-deficient animals [43, 44]. However, the detailed mechanism underlying the role of the complement system in immune-cell recruitment into the brain remains unknown. In the present study, we used complement-deficient mice to evaluate the contributions of the complement component C3 in leukocyte recruitment into the brain during inflammation. Our results demonstrated that C3 plays an essential role in cerebral endothelial activation and contributes to neutrophil recruitment into the brain parenchyma.

Complement activation occurs via three different pathways: classical, alternative, and the mannose-binding lectin (MBL) pathways. As the most abundant component of the complement system, C3 is indispensable for all three pathways of complement activation [45]. The cleavage and activation of C3 initiate the membrane attack pathway, through the formation of the membrane attack complex (MAC), consisting of C5b, C6, C7, C8, and polymeric C9. Neutrophil infiltration into the brain after i.c.v. LPS injection was compromised in C3^−/−^ mice but not in C6^−/−^ mice, suggesting that reduced neutrophil recruitment is not dependent on C6 or MAC assembly. The activation of complement system has broad potential biological consequences, because C3a functions as a chemotactic factor for many types of immune cells [46]. Additionally, we observed significant increased expression of KC(CXCL1) and MIP-2(CXCL2) in the brains of C3^−/−^ mice (data not shown), and this expression likely provides sufficient chemotactic strength for recruiting neutrophils into the brain parenchyma. Therefore, a lack of chemotactic traction does not explain the inhibition of neutrophil recruitment into the CNS in C3^−/−^ mice.

Microglial involvement and endothelial activation are two key steps in recruiting neutrophils into the CNS during LPS-induced inflammation. Microglia are the dominant sentinel cells for the detection of bacterial products, and these cells release TNF-α, which activates the endothelium and facilitates leukocyte rolling, adherence, and recruitment into the CNS parenchyma [47]. In previous studies, we showed that activated glial cells control neutrophil recruitment by secreting TNF-α and CXCL1 [15, 16]. It has recently been reported that C3 is involved in microglial activation and priming under various CNS inflammatory conditions [48]. Additionally, complement C1q and C3 are critical for the innate immune response to *Streptococcus pneumoniae* in the CNS, and C3^−/−^ mice were shown to display reduced expression of IL-1β, IL-12 and MIP-1γ in the CNS in a meningitis animal model [28]. In the present study, only a reduction in the IL-1β level at 24 h was detected in the brain, which is consistent with the findings of previous studies. The levels of KC and MIP-2, essential neutrophil-attracting chemokines, were higher in C3^−/−^ mice than WT mice at 24 h post-LPS injection (data not shown). In contrast, TNF-α, a key cytokine for the stimulation of endothelial activation and subsequent leukocyte recruitment, did not decrease significantly in C3^−/−^ animals. Taken together, these results indicate that C3 deficiency did not affect glial cell activation. Upon LPS stimulation, inflammatory cytokines and chemokines were highly produced in these complement-deficient mice, indicating that reduced recruitment might not reflect the compromised glial activation.

The importance of endothelial cell activation in leukocyte recruitment has been well documented [49, 50]. In a previous study, we reported that a significant increase in TNF-α released from microglia avidly activates the endothelium, causing an increase in adhesion molecule expression and leukocyte recruitment [15]. Upon LPS injection, the glial cells in C3^−/−^ mice produced high levels of TNF-α in the brain parenchyma, although significant reduction in leukocyte rolling and adhesion was observed in the brain microvasculature. Accordingly, the expression levels of adhesion molecules (P-selectin, E-selectin, and VCAM-1), which facilitate leukocyte rolling and adherence in brain postcapillary venules, were downregulated in these mice. Clearly, C3 deficiency affected endothelial activation and significantly reduced E-selectin and VCAM-1 expression in C3^−/−^ animals, even in the presence of high levels of TNF-α, indicating that C3 has synergistic effects with TNF-α on endothelial activation in vivo. C3a stimulates cerebral endothelial activation through its G protein-coupled receptor C3aR. However, TNF-α was able to directly activate endothelial cells to express adhesion molecules in the absence of C3. The mechanism for this discrepancy between the in vivo and in vitro observations remains to be elucidated.

The liver is a primary source of complement components [51], most of which remain within the circulation. Additionally, it has been reported that the local synthesis of complement components in resident cells in the CNS plays an essential role during CNS inflammation [52, 53]. The complement components detected in the CNS are primarily derived from two sources: (1) complement components are locally synthesized from different types of neural glial cells in the brain [54, 55]; and (2) the BBB is not impermeable, and complement components can bypass the barriers and penetrate into the brain parenchyma within 12 h after BBB integrity has been compromised. C3 was only detected in the CNS 12 h after LPS injection. Thus, considering that endothelial cell activation and leukocyte rolling and adhesion were activated at 4 h post-injection, when the C3 level in the brain homogenate was nearly undetectable, C3 from the brain parenchyma was unlikely to facilitate endothelial activation and leukocyte-endothelial interactions. We propose that complement components from the circulation may play a major role in endothelial activation for the following two reasons. First, endothelial activation was dependent on the expression of adhesion molecules on the CNS endothelium. Notably, most of the adhesion molecules were expressed on the luminal side of the CNS endothelium. Thus, it would be easier for C3 in the circulation, rather than factors secreted from the CNS, to mediate the expression of adhesion molecules on the luminal side of the brain endothelium. Second, C3 levels in the circulation were much higher than those in the brain, even after a 100-fold dilution of the plasma samples. Additionally, CVF depletes the complements in the circulation via C3 activation and cleavage. This could potentially cause a massive increase in C3a levels. However, the increase of C3a might not last for a long period and might be depleted from circulation in a very short time. We have already measured levels of C3a at 4 h (the time point for endothelial activation) and 8 h after CVF injection and detected minimum amount of C3a in the circulation. Further, CVF treatment significantly reduced brain endothelial activation. Taken together, these results indicate that it is the C3 in the circulation that facilitates brain endothelial activation.

## Conclusions

Our results showed impaired neutrophil recruitment to the brain vasculature in C3^−/−^ mice and C3aR^−/−^ mice. Endothelial cell activation in these mice was affected by C3 deficiency. Our present study also reveals that the complement component C3a plays a critical role in brain endothelial activation and the expression of adhesion molecules, such as E-selectin and VCAM-1, in response to local LPS administration. Moreover, we provide the first evidence of a synergistic effect between C3 and TNF-α for the activation of brain endothelial cells in vivo. Our findings suggest that blocking or preventing complement activation is a promising approach for suppressing leukocyte recruitment and endothelial activation in inflammatory diseases in the brain.

## Additional files

Additional file 1:
**Time-lapse recordings of the intravascular rolling and adhesion of leukocytes in WT C57BL/6J mice after intraventricular LPS injection (4 h).** Rolling leukocytes were defined as those cells moving at a velocity less than that of erythrocytes. Cells were considered adherent if they remained stationary for at least 30 s in a distance of 100 μm. (MOV 798 kb) Additional file 2:
**Time-lapse recordings demonstrating the lack of the intravascular rolling and adhesion of leukocytes in C3** ^**−/−**^ **mice after intraventricular LPS injection (4 h).** Rolling leukocytes were defined as those cells moving at a velocity less than that of erythrocytes. Cells were considered adherent if they remained stationary for at least 30 s in a distance of 100 μm. (MOV 750 kb) Additional file 3:
**Time-lapse recordings of the intravascular rolling and adhesion of leukocytes in C6** ^**−/−**^ **mice after intraventricular LPS injection (4 h).** Rolling leukocytes were defined as those cells moving at a velocity less than that of erythrocytes. Cells were considered adherent if they remained stationary for at least 30 s in a distance of 100 μm. (MOV 573 kb)

## Abbreviations

BBB: blood-brain barrier
CVF: cobra venom factor
ICAM-1: intercellular cell adhesion molecule 1
i.c.v.: intracerebroventricular(ly)
IL-1β: interleukin-1β
KC: keratinocyte-derived chemokine/CXCL1
KO: knockout
LPS: lipopolysaccharide
MIP-2: macrophage inflammatory protein-2/CXCL2
NF-κB: nuclear factor-κB
PBS: phosphate-buffered saline
TNF-α: tumor necrosis factor-α
VCAM-1: vascular cell adhesion molecule 1
WT: wild-type
CNS: central nervous system
